# Long-term changes in physical fitness components determining the motor performance of young people studying physiotherapy in 2001–2020

**DOI:** 10.1038/s41598-023-41803-0

**Published:** 2023-09-14

**Authors:** Andrzej Lewandowski, Marcin Siedlaczek, Zuzanna Piekorz

**Affiliations:** 1 Chair Department of Physiotherapy Faculty of Health Sciences, Ludwik Rydygier Collegium Medicum in Bydgoszcz Nicolaus Copernicus University, Bydgoszcz, Poland; 2 Department of Basic of Physical Culture, Faculty of Health Sciences, Ludwik Rydygier Collegium Medicum in Bydgoszcz, Nicolaus Copernicus University, Ul. Świetojańska 20, 85-094 Toruń, Bydgoszcz, Poland

**Keywords:** Health care, Health occupations

## Abstract

The aim of the study is to assess changes in basic somatic features and motor components of physical fitness of physiotherapy students in the years 2001–2020. Hypotheses were made about the lack of a secular trend in body height and weight, the deterioration of motor efficiency and the lack of conditioning of the examined changes by social factors. Every year, students of physiotherapy at Collegium Medicum in Bydgoszcz, Nicolaus Copernicus University in Toruń (Poland) were examined in terms of height, weight, chest circumference, balance, speed, power, agility and endurance. The results were obtained from 1161 female students and 464 male students. There was weight gain in the group of women (R^2^ = 0.41, p = 0.00314), deterioration of speed (for women R^2^ = 0.579, p = 0.001; for men R^2^ = 0.301, p = 0.0185) and deterioration of power (for women R^2^ = 0.51, p = 0.001, p = 0.001; for men R^2^ = 0.0432, p = 0.00303). The stability of the remaining features was also found, as well as the lack of their conditioning by social factors. Predictors of maintaining motor fitness were identified, i.e. male gender, fitness exam qualifying for studies and chest circumference. The creation of conditions and requirements encouraging greater care for the appropriate level of motor fitness of people undertaking physiotherapy studies seems justified. This observation may apply to other academia providing training for the profession of physiotherapist.

## Introduction

A physiotherapist is a representative of the medical profession, yet it is based largely on physical activity of both his own and his patients. For many of them, a therapist is the epitome of a physically fit person with a large range of motor skills and professional knowledge that allows them to use their motor attributes in the process of restoring patients' physical fitness^1,2^. From the literature data, it is known that the physical fitness of modern youth differs from that of earlier generations^3,4^. It is commonly believed that it is "worse" because physical activity is successively squeezed out by civilization achievements, which often replace it. There is also an opinion about the changes in its structure as a result of young generations adapting to the requirements of the surrounding reality^5–9^. People studying physiotherapy may also be subject to these processes, especially due to socioeconomic changes and the dynamic development of the profession. This is accompanied by the introduction of curricular changes in studies in this field as well as the qualification requirements for studies^10–12^. The resignation from the fitness exam for physiotherapy studies by most Polish universities is an example of this. The low importance of physical fitness in the successful implementation of the educational process and subsequent occupation is also a disturbing signal for people choosing these studies. From previous research, it is also known that in terms of physical fitness components conducive to the improvement of motor features, people studying physiotherapy at physical education universities have better results on fitness tests and different views on the use of sports for people with disabilities in the therapeutic process^13,14^. It is also known from longitudinal studies that the condition and coordination motor skills of young people studying physiotherapy undergo unfavorable changes^15,16^. There is a high probability that similar changes may occur in other areas of physical fitness. Its components, which are conducive to achieving higher motor efficiency, allow for the successful implementation of therapeutic standards that require physical effort. They also allow for active participation in sports for people with disabilities, which is considered the highest stage of the rehabilitation process. They can also counteract the effects of workloads occurring in this profession^17,18^. The observation of long-term changes in somatic and motor characteristics is all the more important because their secular trends, with varying intensity and directions, still occur in many population groups^8,19,20^.

Therefore, the aim of the undertaken research is to assess changes in the results of fitness tests, which determine the possibility of obtaining motor achievements and basic somatic determinants of people studying physiotherapy at the Medical University of Bydgoszcz in the years 2001–2020, as well as to determine the dependence of the examined features that could cause these changes.

A hypothesis was formulated about the different intensity of the changes in averages of the yearly values of the examined features for women and men and their unfavourable directions for both genders irrespective of sociometric factors, with the exception of performing the fitness exam in the university recruitment process, fitness tests realized in majority which are used to assess the ability to achieve motor results according to the EUROFIT concept^21^. It was also hypothesized that the average value of the Pignet II index will increase and thus testify to changes in the direction of poor physique assessed through the prism of the chest.

## Materials and methods

In the years 2001–2020, the research included young people studying physiotherapy at the Medical University of Ludwik Rydygier in Bydgoszcz (Poland), and after merging with the Nicolaus Copernicus University in Toruń, this was continued at the Collegium Medicum of this university. Bydgoszcz is a city with over 350,000 inhabitants and is the capital of the Kuyavian-Pomeranian region, located in the central-northern part of the country. The data were collected during movement education classes and movement teaching methodology. The course was initially conducted in the third year of uniform master's studies, then in the first and second year, and since 2015, only in the second year of bachelor's studies. Due to the change from master's studies to bachelor's studies in 2014, the observation was not carried out, which from 2019 again included people from master's studies. Until 2010, student groups had to take part in a fitness exam when recruiting for studies. It included a test of swimming skills and measurements of static strength, agility and endurance^22^. With the exception of the group observed in 2020, whose study period took place during the pandemic and lockdown, all academic year groups followed a similar study program. It included 60 h of compulsory physical education, 110 h of movement education with movement teaching methodology, and summer and winter camp activities with a total of 110 teaching hours. Almost all surveyed people graduated from general secondary schools (95.75%), and the vast majority were residents of large cities in the Kuyavian-Pomeranian region in particular (65.97%), with a large part of whom came from Bydgoszcz (20.25%). A significant part of the parents of the surveyed people had secondary education (fathers 35.82%, mothers 42.03%), and a large percentage of them, especially mothers, had higher education (42.58%).

At the end of each academic year covered by the observation, body height and weight were measured as basic somatic features, as well as chest and axilla circumference. Body height was measured with a Swiss GPM anthropometer with an accuracy of 1 mm, and body weight was determined using a Chinese DB-1H Castex electronic scale with an accuracy of 0.01 kg. Body circumferences were measured with an anthropometric measure with an accuracy of 0.1 cm. Their size was used to calculate the Pignet II index, which informs about body build assessed through the prism of the chest^23^.

The Eurofit test was used to examine the components of physical fitness conducive to improving motor performance in the field of general balance (flamingo balance test [FLB]), speed of limb movement (plate tapping [PLT]), explosive strength (standing broad jump [SBJ]), running speed, agility (shuttle run, 10 × 5 m [SHR]) and cardiorespiratory endurance (Cooper test), and their results were recorded in accordance with the test recommendations^21,24^. With the exception of the studies of the last team, whose students themselves carried out possible measurements and fitness tests, all were carried out by a specialist research team under the same conditions and in accordance with the test procedures. The research was carried out with the consent of the Bioethics Committee at Collegium Medicum im. Ludwik Rydygier of the Nicolaus Copernicus University in Bydgoszcz (No. KB 44/2004—extended every 4 years until 2020). All men and women were informed about the purpose of the study, the type and duration of exercise, and the possibility of withdrawing from the study without giving a reason. All procedures contributing to this work followed the ethical standards of the relevant national and institutional human experimentation committees and the 1975 Declaration of Helsinki, amended in 2008. To be included in the research, the following conditions had to be met: being a part of the researched student group, good general condition, and personal informed consent of the participants. The most common exclusion from the study was absence from classes on the day of the measurements or an injury preventing the performance of fitness tests. In total, results were obtained from 464 students and 1161 female students, representing nearly 95% of the surveyed population. The characteristics covered by the research were considered, taking into account the basic sociometric features, such as the size of the place of residence, type of school completed and parents' education.

### Statistics

The results of the research were presented using descriptive statistics in the form of arithmetic averages and standard deviations as well as numerical values and percentages. The relationships between the examined features and the similarity of time changes in the male and female teams were determined by the size of Pearson's correlation coefficients (used to determine the level of dependence between random variables). Changes in score sizes over time were assessed by simple linear regression analysis (used to determine the regression coefficients so as to the model predicts the value of the dependent variable). The analysis of the influence of features on fitness results was carried out using the multiple linear regression method (used to adjust linear or linearized models between one dependent variable and more than one independent variable). The following predictors were used: height and weight, axillary circumference, Pignet II index, fitness exam in the recruitment procedure for studies, gender, age, size of place of residence, type of school completed, father's and mother's education. For each regression model, features were selected according to sequential feature selection "forward" maximizing the R-square index. Statistical analysis was performed at the Statistical Analysis Center of the Nicolaus Copernicus University in Toruń using the Python programming language (3.8.10) with the following libraries: statsmodels (0.13.1), pandas (1.3.4), scikit-learn (1.1.1), matplotlib (3.5.0), and seaborn (0.11.2), setting the significance level alpha = 0.05.

### Ethics

Permission to conduct this study was obtained from the Bioethics Committee of the Ludwik Rydygier Collegium Medicum of Nicolaus Copernicus University in Toruń (no. KB 44/2004). All men and women were informed about the purpose of the study, type and duration of the effort, and the possibility of withdrawing from the study without giving any reason. Each potential participant granted voluntary consent for participation in the study.

## Results

Tables 1 and 2 present characteristics concerning the size of the examined groups of young people and the size of somatic features.

**Table 1:** Demographic characteristics of the participants.

Years of study	n (%)			Age (mean ± SD)		
	All	Men	Women	All	Men	Women
2001	47	12 (25.5)	35 (74.5)	21.55 ± 1.32	22.08 ± 2.27	21.37 ± 0.73
2002	40	13 (32.5)	27 (67.5)	21.12 ± 0.56	21.15 ± 0.69	21.11 ± 0.51
2003	38	6 (15.8)	32 (84.2)	22.79 ± 1.28	23.33 ± 1.37	22.69 ± 1.26
2004	47	8 (17.0)	39 (83.0)	22.32 ± 1.11	23.00 ± 2.14	22.18 ± 0.72
2005	58	11 (19.0)	47 (81.0)	22.10 ± 0.48	22.09 ± 0.30	22.11 ± 0.52
2006	60	8 (13.3)	52 (86.7)	22.25 ± 0.75	23.12 ± 1.55	22.12 ± 0.43
2007	116	37 (31.9)	79 (68.1)	21.65 ± 0.91	21.54 ± 1.02	21.70 ± 0.85
2008	61	21 (34.4)	40 (65.6)	21.59 ± 1.78	22.14 ± 2.83	21.30 ± 0.72
2009	176	69 (39.2)	107 (60.8)	20.99 ± 0.90	21.03 ± 0.94	20.96 ± 0.88
2010	112	33 (29.5)	79 (70.5)	20.40 ± 1.70	20.79 ± 2.51	20.24 ± 1.19
2011	80	28 (35.0)	52 (65.0)	20.36 ± 0.83	20.61 ± 1.20	20.23 ± 0.51
2012	96	30 (31.2)	66 (68.8)	20.33 ± 0.80	20.43 ± 0.90	20.29 ± 0.76
2013	98	31 (31.6)	67 (68.4)	20.40 ± 1.00	20.71 ± 1.35	20.25 ± 0.77
2015	113	30 (26.5)	83 (73.5)	21.30 ± 0.72	21.23 ± 0.57	21.33 ± 0.77
2016	114	26 (22.8)	88 (77.2)	21.25 ± 0.64	21.27 ± 0.60	21.25 ± 0.65
2017	101	26 (25.7)	75 (74.3)	21.19 ± 0.50	21.19 ± 0.40	21.19 ± 0.54
2018	99	23 (23.2)	76 (76.8)	21.24 ± 0.73	21.30 ± 0.93	21.22 ± 0.67
2019	83	26 (31.3)	57 (68.7)	21.37 ± 0.85	21.54 ± 1.03	21.30 ± 0.76
2020	86	26 (30.2)	60 (69.8)	21.34 ± 0.82	21.77 ± 1.07	21.15 ± 0.61

n (%) numerical and percentage characteristics of the size of student groups surveyed; mean—average age of student groups surveyed.

*SD* standard deviation.

**Table 2:** Numerical characteristics of the somatic features of the examined women and men (mean ± SD).

Years of study	Body height (cm)		Body mass (kg)		Chest circumference (cm)		Pignet II index (n)	
	Men	Women	Men	Women	Men	Women	Men	Women
2001	180.70 ± 5.20	165.74 ± 5.15	77.33 ± 10.57	59.11 ± 7.49	94.12 ± 5.73	84.69 ± 5.80	9.24 ± 11.96	21.93 ± 12.11
2002	183.51 ± 5.54	169.25 ± 6.65	75.62 ± 5.39	60.78 ± 7.37	94.23 ± 3.64	82.31 ± 4.66	13.66 ± 10.25	26.16 ± 11.56
2003	182.33 ± 5.30	165.89 ± 7.37	87.17 ± 10.61	59.81 ± 9.78	100.33 ± 5.75	82.41 ± 4.93	-5.17 ± 17.16	23.67 ± 12.11
2004	180.09 ± 3.89	166.07 ± 6.34	80.88 ± 9.42	58.56 ± 9.01	96.75 ± 4.67	82.01 ± 4.64	2.46 ± 15.42	25.50 ± 12.06
2005	175.25 ± 6.40	164.15 ± 5.90	68.64 ± 9.83	59.91 ± 11.44	90.73 ± 7.42	80.56 ± 7.30	15.89 ± 11.03	23.67 ± 15.93
2006	177.38 ± 11.84	165.44 ± 5.07	77.25 ± 13.88	59.60 ± 6.79	96.56 ± 5.05	81.68 ± 4.10	3.56 ± 11.50	24.16 ± 9.69
2007	179.60 ± 5.69	167.82 ± 6.32	75.44 ± 10.05	61.59 ± 9.47	91.78 ± 6.17	81.04 ± 5.41	12.37 ± 15.42	25.18 ± 12.55
2008	180.31 ± 7.18	166.69 ± 5.94	79.00 ± 9.81	58.55 ± 6.73	95.05 ± 6.92	79.62 ± 4.30	6.26 ± 16.15	28.52 ± 9.14
2009	180.99 ± 6.08	166.72 ± 6.23	77.10 ± 10.62	60.36 ± 8.95	93.62 ± 6.65	81.49 ± 5.01	10.26 ± 14.19	24.88 ± 11.80
2010	179.75 ± 6.82	167.61 ± 6.10	76.33 ± 9.52	58.49 ± 7.46	94.06 ± 5.75	81.73 ± 4.41	9.36 ± 10.89	27.38 ± 9.54
2011	177.89 ± 6.84	166.62 ± 4.98	76.64 ± 8.44	60.37 ± 10.05	92.43 ± 4.84	82.24 ± 5.78	8.81 ± 12.52	24.02 ± 15.13
2012	180.45 ± 5.92	166.50 ± 5.87	79.05 ± 15.97	60.49 ± 8.13	93.87 ± 3.95	79.59 ± 4.19	7.53 ± 16.17	26.42 ± 9.78
2013	181.45 ± 6.74	167.97 ± 5.75	76.76 ± 11.47	60.87 ± 9.23	94.23 ± 5.73	80.24 ± 4.50	10.47 ± 11.94	26.87 ± 8.86
2015	179.07 ± 6.63	168.01 ± 6.16	78.67 ± 11.50	61.11 ± 8.00	95.63 ± 6.73	81.57 ± 4.68	4.77 ± 15.75	25.33 ± 11.11
2016	180.85 ± 7.94	165.86 ± 5.52	82.44 ± 15.66	61.14 ± 9.78	97.12 ± 8.96	82.05 ± 6.32	1.30 ± 21.81	22.67 ± 15.12
2017	178.79 ± 5.66	166.18 ± 5.81	81.88 ± 10.87	63.99 ± 11.94	96.19 ± 8.02	82.88 ± 7.34	0.72 ± 17.23	19.30 ± 16.99
2018	179.71 ± 6.70	167.16 ± 5.75	79.72 ± 10.48	61.66 ± 12.03	96.61 ± 6.67	82.70 ± 7.26	3.39 ± 12.18	22.80 ± 18.62
2019	178.63 ± 4.56	166.91 ± 5.75	79.74 ± 12.12	61.92 ± 8.65	97.03 ± 8.20	82.50 ± 5.44	1.86 ± 19.08	22.49 ± 11.81
2020	183.23 ± 5.38	168.15 ± 5.76	84.93 ± 12.86	61.09 ± 10.07	104.38 ± 9.81	86.89 ± 6.84	-6.09 ± 20.27	20.16 ± 14.29

*SD* standard deviation.

The characteristics of the descriptive statistics indicate that in all years of observation, women accounted for significantly larger shares with slightly lower average ages and standard deviations.

The men covered by the observation were characterized by higher mean somatic features and mean standard deviations, as well as lower average values of the Pignet II index, indicating a strong build contrasting with a weak build of women.

Tables 3 and 4 show the results of the fitness tests of men's and women's teams.

**Table 3:** Numerical characteristics of the results of fitness tests of the examined men (mean ± SD).

Years of study	General balance (n)	Speed of limb movement (n)	Explosive strength (cm)	Running speed, agility (n)	Cardio-respiratory endurance (m)
2001	7.50 ± 3.48	90.25 ± 6.58	236.58 ± 17.57	196.50 ± 14.93	2645.50 ± 385.31
2002	7.15 ± 4.14	94.23 ± 6.17	243.62 ± 15.64	196.38 ± 10.40	2552.46 ± 276.42
2003	10.33 ± 2.34	99.00 ± 17.29	229.33 ± 15.46	190.33 ± 8.82	2339.17 ± 342.59
2004	9.75 ± 3.99	96.62 ± 12.67	227.00 ± 20.54	197.00 ± 9.77	2322.12 ± 373.94
2005	8.36 ± 3.32	89.09 ± 11.93	233.45 ± 27.21	182.09 ± 15.69	2370.91 ± 508.31
2006	10.38 ± 2.62	97.12 ± 18.67	221.25 ± 20.83	198.88 ± 10.96	2268.75 ± 279.10
2007	8.08 ± 2.92	88.70 ± 12.08	233.43 ± 16.66	193.57 ± 24.64	2214.30 ± 365.48
2008	7.76 ± 4.35	89.43 ± 16.36	232.10 ± 16.24	198.48 ± 22.91	2574.90 ± 381.99
2009	8.51 ± 3.48	92.91 ± 10.93	241.12 ± 18.49	184.29 ± 11.08	2660.88 ± 333.01
2010	8.12 ± 3.43	92.76 ± 9.16	232.06 ± 16.03	190.03 ± 11.89	2686.48 ± 261.75
2011	8.25 ± 3.42	95.07 ± 14.19	215.39 ± 22.96	192.50 ± 18.44	2488.64 ± 353.53
2012	7.73 ± 4.41	92.67 ± 9.50	223.07 ± 28.32	175.97 ± 20.84	2641.07 ± 373.10
2013	7.16 ± 4.53	99.03 ± 12.25	218.87 ± 20.47	205.10 ± 23.00	2387.10 ± 368.33
2015	9.83 ± 5.09	95.43 ± 13.55	220.23 ± 25.84	204.60 ± 23.26	2471.27 ± 365.53
2016	10.27 ± 3.41	100.96 ± 12.61	221.77 ± 20.56	197.00 ± 15.59	2381.12 ± 299.85
2017	8.58 ± 4.67	100.23 ± 20.30	213.27 ± 23.16	215.65 ± 32.33	2376.23 ± 340.29
2018	10.17 ± 4.93	98.30 ± 16.83	215.00 ± 24.64	202.13 ± 23.62	2387.61 ± 338.45
2019	7.96 ± 4.75	99.69 ± 12.81	229.00 ± 28.13	194.54 ± 19.56	2399.42 ± 370.01
2020	-	-	-	-	2575.88 ± 356.12

*SD* standard deviation.

**Table 4:** Numerical characteristics of the results of fitness tests of the surveyed women (mean ± SD).

Years of study	General balance (n)	Speed of limb movement (n)	Explosive strength (cm)	Running speed, agility (n)	Cardio-respiratory endurance (m)
2001	6.74 ± 3.72	98.31 ± 7.90	181.20 ± 17.15	213.86 ± 12.69	2046.03 ± 197.10
2002	6.78 ± 2.76	99.70 ± 6.70	188.26 ± 16.35	209.81 ± 15.21	2227.15 ± 257.68
2003	10.62 ± 3.41	105.69 ± 23.29	174.59 ± 19.95	208.19 ± 10.46	1882.78 ± 272.72
2004	9.59 ± 4.96	100.46 ± 11.25	178.97 ± 20.53	214.74 ± 16.04	1997.33 ± 345.48
2005	9.19 ± 4.50	100.23 ± 12.01	180.94 ± 18.77	200.17 ± 15.82	1937.45 ± 287.71
2006	9.15 ± 3.79	101.60 ± 11.92	177.69 ± 18.34	207.13 ± 17.12	1818.48 ± 233.39
2007	9.00 ± 3.29	103.62 ± 11.97	185.01 ± 19.09	206.80 ± 13.80	1907.91 ± 356.33
2008	7.42 ± 3.06	100.92 ± 12.90	179.62 ± 18.11	205.45 ± 12.36	2119.88 ± 276.88
2009	8.68 ± 4.27	100.54 ± 12.57	185.56 ± 19.60	198.07 ± 19.65	2145.77 ± 255.08
2010	9.57 ± 3.41	100.01 ± 9.30	184.62 ± 18.05	206.30 ± 11.22	2193.30 ± 178.29
2011	10.23 ± 4.48	107.90 ± 14.18	169.83 ± 16.96	205.35 ± 13.32	2148.87 ± 225.38
2012	8.14 ± 3.51	104.97 ± 12.69	175.14 ± 17.53	185.23 ± 25.67	2211.09 ± 366.38
2013	9.97 ± 4.75	108.21 ± 10.10	173.06 ± 16.32	216.51 ± 15.11	1987.10 ± 275.71
2015	8.80 ± 4.73	112.54 ± 16.67	171.00 ± 19.70	214.17 ± 15.18	2077.02 ± 235.61
2016	7.81 ± 3.37	111.56 ± 19.67	173.59 ± 18.03	211.92 ± 18.74	2183.95 ± 260.23
2017	9.04 ± 4.08	107.63 ± 15.81	169.27 ± 21.66	220.95 ± 20.48	1927.61 ± 260.94
2018	9.91 ± 5.33	109.05 ± 20.32	165.97 ± 20.35	221.07 ± 35.44	1999.54 ± 261.02
2019	8.00 ± 3.64	105.72 ± 12.95	172.04 ± 17.09	203.81 ± 29.81	1991.77 ± 214.29
2020	–	–	–	–	2102.78 ± 264.30

*SD* standard deviation.

The examined men, with the exception of the balance test, in which the averages of the teams of both genders had similar values, were characterized by higher average fitness test results and lower average speed of hand movements and the running test, which also means a better result. The size of the standard deviations of the results of the men's and women's teams took different directions.

Table 5 presents a single picture of the size of the Pearson correlation coefficients of the average values for individual features from all the years studied, depending on the selected combination.

**Table 5:** Numerical characteristics of the relationship between the examined features.

Age	1									
Body height	2	− 0.01								
Body mass	3	**0.08**	**0.68**							
Chest circumference	4	**0.09**	**0,62**	**0.85**						
Pignet II index	5	− **0.12**	− **0.34**	− **0.89**	− **0.88**					
General balance	6	0.02	0.03	**0.14**	**0.08**	− **0.14**				
Speed of limb movement	7	0.02	− **0.22**	− **0.18**	− **0.22**	**0.14**	**0.14**			
Explosove strength	8	0.01	**0.59**	**0.36**	**0.47**	− **0.22**	− **0.20**	− **0.07**		
Running speed, agility	9	0.04	− **0.17**	− **0.08**	− **0.12**	0.03	**0.15**	**0.23**	− **0.40**	
Cardio-respiratory endurance	10	− **0.11**	**0.41**	**0.17**	**0.28**	− **0.06**	− **0.19**	− **0.20**	**0.57**	− **0.33**
Factors	nr	1	2	3	4	5	6	7	8	9

Correction factors of the examined features.

Bold indicates statistically significant correlations p < 0.05.

As seen from the list, the greatest correlations were found between somatic characteristics, as well as between these characteristics and the results of the standing long jump. The highest correlation coefficients of motor features were found between the results of the Cooper test and the long jump and were negative with the results of the shuttle run, which was also negatively correlated with the results of the long jump. The remaining Pearson correlation coefficients, which showed statistically significant relationships, did not exceed the value of 0.30.

The relationship of changes in the average annual values of the examined characteristics between women and men was assessed with Pearson's linear correlation coefficients, which are presented in Table 6.

**Table 6:** Evaluation of the relationship between changes in average annual values of female and male characteristics.

Factors	Pearson's linear correlation coefficient	p
Age	**0.909**	**0.001**
Body height	**0.612**	**0.001**
Body mass	0.190	0.436
Chest circumference	**0.656**	**0.002**
Pignet II index	**0.516**	**0.024**
General balance	0.430	0.075
Speed of limb movemend	**0.633**	**0.005**
Explosive strength	**0.899**	**0.001**
Running speed, agility	**0.888**	**0.001**
Cardio-respiratory endurance	**0.782**	**0.001**

Correlation coefficient linear Pearson of the tested features.

Bold indicates statistically significant correlations p < 0.05.

Pearson's linear correlation coefficients, with the exception of body weight and general balance, are statistically significant for the other characteristics covered by the observation. This means that changes in the characteristics covered by the observation were in clear majority similar among women and men.

The dependence of the studied features on time was assessed using a simple linear regression model whose coefficients for women are presented in Table 7 and for men in Table 8.

**Table 7:** Coefficients of linear regression models for female characteristics.

	Coef	P > |t|	[0.025	0.975]
Age (R-kwadrat = 0.173)				
Const	117.948	0.034	9.716	226.179
× 1	− 0.048	0.077	− 0.102	0.006
Body height (R-kwadrat = 0.066)				
Const	65.266	0.491	− 130.475	261.007
× 1	0.051	0.289	− 0.047	0.148
Body mass (R-kwadrat = 0.41)				
Const	− **230.364**	**0.014**	− **408.887**	− **51.842**
× 1	**0.145**	**0.003**	**0.056**	**0.233**
Chest circumference (R-kwadrat = 0.049)				
Const	− 42.050	0.756	− 323.227	239.127
× 1	0.062	0.365	− 0.078	0.202
Pignet II index (R-kwadrat = 0.155)				
Const	337.681	0.074	− 36.660	712.022
× 1	− 0.156	0.095	− 0.342	0.030
General balance (R-kwadrat = 0.024)				
Const	-53.327	0.596	− 262.480	155.826
× 1	0.031	0.538	− 0.073	0.135
Speed of limb movement (R-kwadrat = 0.579)				
Const	− **1070.875**	**0.001**	− **1602.071**	− **539.679**
× 1	**0.585**	** < 0.001**	**0.320**	**0.849**
**Explosove strength (R-kwadrat = 0.51)**				
const	**1787.784**	**0.000**	**951.046**	**2624.521**
× 1	− **0.801**	**0.001**	− **1.218**	− **0.385**
Running speed, agility (R-kwadrat = 0.029)				
Const	− 309.836	0.684	− 1894.106	1274.433
× 1	0.258	0.498	− 0.530	1.046
Cardio-respiratory endurance (R-kwadrat = 0.017)				
Const	− 3233.886	0.744	− 23,800.000	17,300.000
× 1	2.627	0.595	− 7.603	12.857

coef—linear regression model coefficient of the variable.

P > |t|—p-value of the linear regression model coefficient.

[0.025–0.975] – upper and lower bounds of the 95% confidence interval for the linear model coefficient.

× 1—year.

Statistically significant models are marked with bold p < 0.05.

**Table 8:** Coefficients of linear regression models for male characteristics.

	Coef	P > |t|	[0.025	0.975]
Age (R-kwadrat = 0.231)				
Const	**157.496**	**0.018**	**30.512**	**284.481**
× 1	**− 0.068**	**0.037**	**− 0.131**	**− 0.004**
Body height (R-kwadrat = 0.0)				
Const	194.147	0.245	− 145.658	533.952
× 1	− 0.007	0.931	− 0.176	0.162
Body mass (R-kwadrat = 0.12)				
Const	− 380.629	0.224	− 1016.516	255.258
× 1	0.229	0.146	− 0.088	0.545
Chest circumference (R-kwadrat = 0.154)				
Const	− 308.867	0.197	− 793.758	176.023
× 1	0.201	0.096	− 0.040	0.442
Pignet II index (R-kwadrat = 0.197)				
Const	883.644	0.056	− 23.643	1790.930
× 1	− 0.437	0.057	− 0.888	0.015
General balance (R-kwadrat = 0.022)				
Const	− 51.495	0.611	− 262.065	159.076
× 1	0.030	0.553	− 0.075	0.135
Speed of limb movement (R-kwadrat = 0.301)				
Const	− **687.505**	**0.035**	− **1320.021**	− **54.988**
× 1	**0.389**	**0.018**	**0.075**	**0.704**
Explosove strength (R-kwadrat = 0.432)				
Const	**2303.467**	**0.001**	**1041.986**	**3564.948**
× 1	− **1.033**	**0.003**	− **1.661**	− **0.405**
Running speed, agility (R-kwadrat = 0.117)				
Const	− 900.244	0.250	− 2497.408	696.920
× 1	0.545	0.165	− 0.250	1.340
Cardio-respiratory endurance (R-kwadrat = 0.0)				
Const	2513.271	0.829	− 21,700.000	26,700.000
× 1	− 0.026	0.996	− 12.065	12.012

coef—linear regression model coefficient of the variable.

P > |t|—p-value of the linear regression model coefficient.

[0.025–0.975] – upper and lower bounds of the 95% confidence interval for the linear model coefficient.

× 1—year.

Statistically significant models are marked with bold p < 0.05.

For the age of women, the model is not significant and indicates that the value of the feature is maintained at a similar level. For the age of men, the model is significant and indicates a decrease in the average age of students along with the progressive change of the surveyed academic year groups by 0.068 years.

For body height, as well as axillary circumference and Pignet II index in women and men, the models are not significant and indicate that the values of the features are maintained at a similar level.

For women's body weight, the model is significant and indicates an increase in the average value of this feature year-on-year by 0.145 kg. For the body weight of men, the model is not relevant.

For the results of the general balance test, as well as the pendulum run and the Cooper test of women and men, the models are not significant and indicate that the values of the features are maintained at a similar level.

For the results of the female hand speed test, the model is significant and indicates an increase in the average value of this test year-on-year by 0.585 measurement units. For the results of men, the model is also important and indicates an increase in the average value of this sample year on year by 0.389 measurement units.

For women's long jump results, the model is significant and indicates a decrease in the average value of this test year-on-year by 0.801 cm. For the results of men, the model is also significant and indicates a decrease in the average value of this sample year-on-year by 1.033 cm.

The model of multiple linear regression was also used to check which of the somatic and sociometric characteristics adopted for the analysis significantly affect the results of fitness tests, provided that all other indicators are the same. The most important features were selected using the sequential feature selection "forward" method, maximizing the R-square index. Its value and the level of significance of the regression models ≤ 0.001 determine the predictors, which are presented in Table 9.

**Table 9:** Coefficients of significant linear regression models for the motor characteristics of the tested team.

	Coef	P > |t|	[0.025	0.975]
General balance				
Const	3.524	0.001	2.276	4.772
Body mass	0.088	0.001	0.068	0.108
Male	− 1.905	0.001	− 2.472	− 1.338
Speed of limb movement				
Const	108.454	0.001	107.378	109.530
Fitness exam	− 7.012	0.001	− 8.417	− 5.608
Male	− 10.162	0.001	− 11.718	− 8.606
Explosove strength				
Const	49.978	0.001	21.627	78.329
Chest circumference	1.247	0.001	0.934	1.559
Pignet II index	0.794	0.001	0.660	0.929
Fitness exam	11.284	0.001	9.388	13.181
Male	48.252	0.001	45.094	51.411
Running speed, agility				
Const	191.517	0.001	184.928	198.105
Body mass	0.275	0.001	0.168	0.382
Male	− 18.601	0.001	− 21.594	− 15.608
Cardio-respiratory endurance				
Const	1275.904	0.001	773.614	1778.194
Chest circumference	15.223	0.001	10.704	19.743
Pignet II index	11.543	0.001	9.530	13.555
Age	− 35.337	0.001	− 48.197	− 22.476
Male	441.987	0.001	394.859	489.114

coef—linear regression model coefficient of the variable.

P > |t|—p-value of the linear regression model coefficient.

[0.025–0.975] – upper and lower bounds of the 95% confidence interval for the linear model coefficient.

For general balance, R-square = 0.047, p = 7.31e-17, and the model explains the variability in 4.7% with the significance of the whole model p = 0.001. An increase in the body weight variable by one unit increases the balance score by 0.0883 points, which means its deterioration, and male sex reduces the score by -1.9051 points, which means an improvement in the test result.

For hand speed, R-square = 0.146, p = 2.5499999999999998e−53, and the model explains 14.6% of the variability with the significance of the whole model p = 0.001. The physical fitness test causes a decrease in the result by − 7.0121 points, and the male sex by − 10.1623 points, which in both cases means an improvement in the result of the test.

For the explosive strength, R-square = 0.629, p = 0.0, and the model explains 62.9% of the variability with the significance of the whole model p = 0.001. Increasing the axillary circumference by 1 cm extends the standing long jump by 1.2466 cm, and increasing the Pignet II index by 1 increases its distance by 0.7943 cm. To a much greater extent, the result of this characteristic is influenced by the presence of a fitness exam during recruitment for studies, which increases the result by 11.2844 cm, and the male gender extends it by 48.2523 cm.

For agility, R-square = 0.094, p = 1.67e−33, and the model explains 9.4% of the variability with the significance of the whole model p = 0.001. An increase in body weight by 1 kg results in an increase in the result by 0.2751, which means a deterioration of the test result, and male sex decreases it by − 18.6008, showing an improvement in the result.

For strength R-square = 0.361, p = 1.43e−155, and the model explains the variability in 36.1% with the significance of the whole model 0.001. Increasing the axillary circumference by 1 cm improves the running result by 15.2234 m, and the Pignet II index increases by 11.5427 m. To a much greater extent, the change in the result of the Cooper test is influenced by age, which, when increased by a year, shortens the distance traveled by 35.3367 m, and the male gender extends it by 441.9866 m.

As seen from the summary, body height and sociometric characteristics, i.e. The size of the place of residence, type of school completed and parents' education did not affect the maintenance of the level of the observed motor characteristics.

## Discussion

The presented results of long-term studies of motor changes in people studying physiotherapy provided valuable information, which with high probability can be a picture of the situation in the Polish population of young healthy people, where future physiotherapists from Bydgoszcz are characterized by a satisfactory assessment of physical fitness^25^. The obtained results, however, must be referenced to the available literature data, making the necessary comparisons and using them to assess the accuracy of the hypotheses.

The main observation is the continuing predominance of women in physiotherapy studies, which, despite the decreasing trend, is still a phenomenon typical for medical professions^26^. In the case of the profession of a physiotherapist, such a situation seems to be somewhat disturbing in the context of the need for therapists to adopt the standards that require physical effort, which the results of fitness tests being worse than those of men do not predispose the surveyed woman to. This state of affairs, especially in terms of manifestations of fitness abilities, is consistent with the basic literature knowledge^27,28^. Similar results of the general balance test of the examined people of both sexes, which is a component of motor coordination, despite the center of gravity in women being located lower, which is conducive to obtaining a more favorable result, proves that its level is largely conditioned by other factors, including the efficiency of the nervous system, which thus seems to be greater in the men studied. The above assumption justifies the observed decrease in the level of rotational coordination of women in both decades covered by the observation and in the case of men only to the left in the second decade^15,16^. In their case, it also seems beneficial to reduce the average age and bring it closer to that of female students. This may mean that over time, physiotherapy studies are becoming the first choice of education for the future profession for an increasing number of men, which as a result of many changes is becoming more socially and economically attractive, as we have already suggested in previous studies^15,16^.

Interesting information was also provided by the analysis of the size of the correlation coefficients of the observed features, which shows that the greatest relationships occur between the examined somatic features and between these features and explosive power, as well as explosive power and endurance. This proves that body build largely determines motor efficiency, especially since the examined determinants of somatic features correlate with tests of a high accuracy coefficient concerning symptoms significant in obtaining motor achievements^21,24,29,30^.

The analysis of the material concerning the course of changes in the examined characteristics of men and women, with the exception of body weight and general balance, showed a great similarity between them. Thus, it indicates a similar impact of the burdens related to the implementation of studies and student's life on the somatic and motor sphere of people of both sexes. We believe that the differences in the course of changes in body weight in the examined groups are related to its increase in the group of women and small changes in the examined men, and the lack of similarity in the course of changes in the level of general balance probably results from the stability of this characteristic in the teams of both sexes over the decades covered by the observation. In this regard, the hypothesis about the lack of similarity in changes in the average annual values of the observed characteristics between men and women was negatively verified.

However, the main objective of the study was to determine the magnitude of changes in the examined features over the period of two decades of observation. In terms of basic somatic features, a secular trend in women's body weight and its absence in the body height of the teams of both sexes were found, as well as the stability of the axillary circumference and body build assessed through prism was observed. These results are in contradiction with the results of long-term observations of students from Zielona Góra, which found that the secular trend in height and weight of both sexes persisted^31^. Therefore, we believe that there may be a slow change in body structure among women undertaking physiotherapy studies. However, it should be noted that the Pignet II index is based on a small number of somatic features, and thus, the assessment of body build based on its size has a relative value.

In terms of the observed manifestations of motor skills in the teams of both sexes, the stability of general balance, agility and endurance were found. This result corresponds to the aforementioned studies, in which a regression of most components of physical fitness conducive to motor achievements was found^31^. In this respect, the hypothesis of negative changes in motor efficiency was verified negatively in contrast to the changes in the speed of hand movements and explosive power, the results of which deteriorated. This observation is somewhat concerning because the therapeutic standards of a dynamic nature are professional activities that significantly overload the bodies of physiotherapists^32^. The deterioration of the speed of hand movements indicates a decrease in the level of speed, the level of motor coordination, and thus the efficiency of the nervous system, which does not correspond to the expected contemporary manifestation of close movements and rapid reaction of the nervous system^6^. We also assume that the stability of the results of running tests may be the result of the fashion for practicing a running activity, which has prevailed among young people for many years. However, the lack of significant changes in endurance measured by the Cooper test among women may be the result of improving results in the first decade and its worsening in the second decade^15,16^. The continuation of such a trend is therefore a worrying prognosis, especially since the currently identified negative changes in the components of physical fitness supporting motor achievements may also result from the resignation of the fitness exam in the recruitment process in the second decade and previous physical experience, as well as the lack of sports activity by a large proportion of people before and during their studies. It is also possible that young people undertaking physiotherapy studies limit or identify their own physical activity with later clinical activity, which is increasingly carried out as part of apprenticeships included in the study programme^33,34^. This type of attention to physical fitness differs significantly from that in sports activity and certainly does not bring the same effects. Our far-reaching supposition, however, requires verification by separate and broader studies of the pro-health behaviors of students^35^. As a summary of the results of the part of the research on changes in long-term manifestations of motor skills of people studying physiotherapy, we consider the statement that no significant improvement in the level of any of the components of physical fitness supporting motor achievements was observed over the period of two decades. However, there was a trend of deterioration in the level of general balance and agility as well as endurance of men, and significant negative changes were identified in the area of speed and explosive power of both sexes, which are very important in achieving motor achievements^29,30^. Their great importance was known even in ancient times, including the standing long jump with the rejection of weights to the pentathlon, which was considered the most important competition of the ancient games.

In the research concerning the maintenance of the results of fitness tests, the corresponding predictors were identified. Male sex turned out to be the expected one, as it improves the level of all tested motor characteristics, which is consistent with the available literature data^27,28,30^. The influence of the axillary circumference and the Pignet II index on the improvement of endurance and explosive power also turned out to be expected. The shape of the chest, to some extent, may be the result of undertaking the physical activity, and thus, it is possible that the lack of significant changes in the axillary circumference and Pignet II index, as highly correlated features, results from similar physical activity undertaken by the examined teams during the observation period^36^. However, the accuracy of this thesis should be supported by anthropometric studies of the chest and motor behavior of students. The impact of body weight on the deterioration of general balance and agility, as well as age, the increase of which deteriorates the level of endurance, corresponds to the available literature^28,30,37^. Such research results show that the components of motor fitness of people studying physiotherapy are conditioned to varying degrees by morphological factors, and basic sociodemographic factors do not significantly affect their level. This indicates the possibility of the effects of indirect, perhaps intellectual, selection for studies and thus its more "sharp" impact among people from lower levels of social class. A somewhat surprising result of the research was the impact of the presence of a fitness exam in the recruitment procedure for studies on the examined components of motor fitness, which determined the improvement only in the explosive strength and speed of hand movements. Their results in the observation period significantly deteriorated, which could be partly due to the resignation of the fitness test in the second decade of research. Thus, the hypothesis of the impact of sociodemographic and somatic factors on the results of fitness tests was fully positively verified only in relation to the feature—male sex, and the remaining features were predictors of no more than two out of five examined manifestations of motor skills of people covered by the observation.

The analysis of the obtained results encountered limitations related to the small number of publications of studies on similar topics, the results of which could be used as comparative material, making the current observation credible. The presented study, therefore, does not exhaust the issues of long-term changes in motor fitness of people studying physiotherapy, but within the scope of observation, they may reflect trends also occurring in other academic environments providing education in this field, thus giving the possibility of a wider use of the following inference.

## Conclusions

The obtained results, which allow the observation of changes in the components of physical fitness supporting motor achievements occurring in the years 2001–2020 in the Bydgoszcz population of physiotherapy students, showed their partial stability and significant deterioration of the level of the most important ones, as well as the lack of their conditioning by basic social factors. Thus, the creation of conditions and requirements encouraging this group of young people to take greater care of the appropriate level of motor fitness seems justified, especially since the observation made may also apply to other academic environments providing training for the profession of physiotherapist.

## Data Availability

The datasets used and/or analysed during the current study are available from the corresponding author on reasonable request.
